# Mathematical modeling of transdermal delivery of topical drug formulations in a dynamic microfluidic diffusion chamber in health and disease

**DOI:** 10.1371/journal.pone.0299501

**Published:** 2024-04-11

**Authors:** Gábor Szederkényi, Dorottya Kocsis, Mihály A. Vághy, Domonkos Czárán, Péter Sasvári, Miléna Lengyel, Márton Bese Naszlady, Fabiola Kreis, István Antal, Roland Csépányi-Kömi, Franciska Erdő

**Affiliations:** 1 Faculty of Information Technology and Bionics, Pázmány Péter Catholic University, Budapest, Hungary; 2 Systems and Control Laboratory, HUN-REN Institute for Computer Science and Control (SZTAKI), Budapest, Hungary; 3 Department of Physiology, Semmelweis University, Budapest, Hungary; 4 Department of Pharmaceutics, Semmelweis University, Budapest, Hungary; Queen’s University Belfast, UNITED KINGDOM

## Abstract

Mathematical models of epidermal and dermal transport are essential for optimization and development of products for percutaneous delivery both for local and systemic indication and for evaluation of dermal exposure to chemicals for assessing their toxicity. These models often help directly by providing information on the rate of drug penetration through the skin and thus on the dermal or systemic concentration of drugs which is the base of their pharmacological effect. The simulations are also helpful in analyzing experimental data, reducing the number of experiments and translating the in vitro investigations to an in-vivo setting. In this study skin penetration of topically administered caffeine cream was investigated in a skin-on-a-chip microfluidic diffusion chamber at room temperature and at 32°C. Also the transdermal penetration of caffeine in healthy and diseased conditions was compared in mouse skins from intact, psoriatic and allergic animals. In the last experimental setup dexamethasone, indomethacin, piroxicam and diclofenac were examined as a cream formulation for absorption across the dermal barrier. All the measured data were used for making mathematical simulation in a three-compartmental model. The calculated and measured results showed a good match, which findings indicate that our mathematical model might be applied for prediction of drug delivery through the skin under different circumstances and for various drugs in the novel, miniaturized diffusion chamber.

## 1 Introduction

To simulate drug penetration and diffusion across the physiological barriers several methods have been described [1–5]. However, these in silico methods mainly focused on molecular docking. Faragó and coworkers were devoted to obtaining the molecular, electronic, and vibrational data for tacrolimus by using five semi-empirical methods and one Density Functional Theory method to get knowledge about the drug properties by computational simulation [1]. To test novel topical anandamide formulation for alleviating peripheral neuropathic pain Police and coworkers used also molecular docking methods and simulation [2]. The compartmental pharmacokinetic models (Physiologically-Based Pharmacokinetic Models) are used by other authors with a focus on blood-brain barrier/ blood-CSF-barrier penetration [6, 7], or e.g. intestinal absorption [8]. Guy and Hadgraft reported a physically based pharmacokinetic description of skin absorption in some early articles [9–12]. The effect of penetration enhancers on the kinetics of percutaneous absorption was also studied by these authors [13]. However, in the later time less attention was paid to compartmental simulation of drug delivery across the percutaneous barrier.

Substances applied to the surface of the skin reach the dermal microcirculation by penetration through the cells of the dead and viable epidermis (transcellular route), between the cells of the epidermis (paracellular route), and through the skin appendages (hair follicles and sweat glands) (transappendageal route). Penetration through the uppermost keratinized layer, the stratum corneum, is the rate-limiting step in skin absorption while the viable epidermis and dermis offer little resistance to penetration [14, 15]. The skin appendages act as shunts–parallel penetration pathways that bypass the route through the epidermal cells. The interappendageal route has been visualized qualitatively by histochemical and autoradiographic techniques [16]. However, specific quantitation of transcellular and appendageal penetration has rarely been reported. Although the small total area of skin appendages minimizes their contribution to transport, shunt penetration may be significant, especially before steady state is established [17]. Middleton [18] has suggested that electrolytes penetrate paracellularly in the stratum corneum, while Blank et al. [19] proposed that water and other polar molecules penetrate through the aqueous regions between the macromolecular components of the epidermal cell membranes. Scheuplein and Blank concluded that “the precise contribution of the appendages to the total drug delivery cannot be calculated for a system as complicated and variable as the skin” [15].

During the last decades the traditional Franz diffusion cells were widely used for testing drug absorption through the skin barrier. However, these systems usually did not take into account the dynamic movements of the fluids in extracellular matrix and the dermal microvasculature. To mimic the shear stress, which greatly influences the topical drug penetration, miniaturized dynamic diffusion cells were designed and fabricated by several research groups. These so called “skin-on-a-chip” microfluidic systems create a physiologically more relevant environment to study drug release and absorption after application on the skin surface. Although, the miniaturized devices require much less animal or human tissue, than the traditional diffusion chambers, but still expect natural tissue. The aim of our simulation study was to provide evidence, that our mathematical model is able to substitute the ex vivo “skin-on-a-chip” experiments. Or at least this model can provide reliable data for prediction of drug, cosmetic or industrial material penetration into or across the dermal barrier. For this purpose, the experimental data acquired from ex vivo skin-on-a-chip diffusion experiments using abdominal mouse skins treated with 2% caffeine or NSAIDs and SAID containing creams under various conditions, were compared with the data calculated by mathematical pharmacokinetic modelling for similar situations. The influence of temperature, sample collection time, characteristics of the test compounds and the integrity of skin barrier in health and dermatological disorders (psoriasiform dermatitis and allergic contact dermatitis) were investigated. To gain basic information about the tested drug formulations, rheological and particle size distributions studies were also conducted.

Mathematical modeling and simulation can efficiently support the analysis of epidermal and dermal transportation processes and also the design and evaluation of experiments and devices for testing percutaneous drug delivery [20, 21]. Since the physical processes of the change of drug concentrations in different sections of the skin are well-known [22], a straightforward choice is to use differential equations for modeling. The first step in modeling is the derivation of an appropriate partial differential equation (PDE) starting from a diffusion equation, where the unknown is the substance concentration as a function of space and time [23, 24].

Then it is practical to spatially discretize the PDE to obtain a set of ordinary differential equations (ODEs) the complexity of which depends on the modeling goal. A physically meaningful (e.g., nonnegative, conservative) discretization gives a so-called compartmental model, where the compartments correspond to various layers of the skin. The theory and application of compartmental models is an intensively studied area with strong theoretical results and efficient computation methods [25, 26]. Model calibration using experimental data is often a challenging task for biological models [27, 28]. The first fundamental problem is the theoretical possibility to determine each unknown model parameter from the available measurements which are assumed to be perfect (i.e., entirely known and noiseless). If the computation of parameters is mathematically possible then the model is called structurally identifiable [29]. It is known that deciding structural identifiability of general nonlinear models is a computationally hard task. However, there exist several efficient methods and corresponding software tools for checking the structural identifiability of dynamical models based on e.g., differential algebra, the power-series expansion of the observed output, or observability [30–34]. Moreover, the special properties of compartmental models can be efficiently used in identifiability analysis [35, 36]. Even if a model is structurally identifiable, the (statistical) quality of the calibration might not be satisfactory due to the limited information content of the measurements. This is often called a practical identifiability problem [37]. Clearly, the solution of identifiability problems often requires the re-iteration of the modeling and/or the experiment design process.

## 2 Materials and methods

### 2.1 Caffeine and anti-inflammatory drugs

Caffeine was used as a hydrophilic model drug for testing skin permeability under different conditions. Caffeine was purchased from Sigma Hungary Kft (Budapest, Hungary). To compare the absorption of topically administered anti-inflammatory drugs, one steroidal (dexamethasone-acetate) and three non-steroidal (diclofenac, indomethacin, piroxicam) anti-inflammatory molecules were formulated as O/W creams. Dexamethasone-acetate is widely used in the human therapy in different formulations like tablets, solutions (eye drops and injections), ointments and intravitreal implants. Also in preclinical in vitro and in vivo studies dexamethasone is a popular steroidal model drug. Indomethacin, piroxicam and diclofenac are strong acting active NSAIDs, that are widely marketed. They have many topical (oinments, gels, patches, solutions, suspensions) and systemic (suppositoris, tablets, injections, capsules, granulates, suspensions) formulations in the market. These drugs are also frequently applied in preclinal studies (subsection 2.2). The active substances were purchased from Sigma Hungary Kft (Budapest, Hungary). For diffusion studies a peripheral perfusion fluid was prepared (PPF). It has the following composition: 147 mM NaCl, 4 mM KCl and 2.3 mM CaCl_2_, all substances were acquired from Sigma-Hungary Kft., Budapest, Hungary. pH of peripheral perfusion fluid was 7.4.

### 2.2 Cream formulations

The creams were prepared with the following composition: 46% Petrolatum basis ointment (which is composed of 26% Vaselinum album, 12% Alcohol cetylicus et stearilycus (Molar Chemicals Kft., Halásztelek, Hungary), 10% Propyleneglycolum, 8% Paraffinum liquidum, 4% Polysorbate 60, and 40% Aqua purificata), 4% Paraffin oil, 38% Purified water and 10% Propylene glycol and 2% active pharmaceutical ingredient. The ingredients of the creams were purchased from Hungaropharma Zrt, Budapest, Hungary except other is indicated.

The creams were prepared ex tempore under magistral conditions. The lipophilic components were melted in an enamel bowl over a water bath, Polysorbate 60 was added, homogenized with a pestle, and then the water of the same temperature was emulsified in the lipophilic phase. The cream was stirred continuously, and the active ingredient (2%) was dispersed thoroughly in it. The preservative was added to the preparation when it cooled below 30°C and stirring was continued until the cream was cooled further to room temperature. The prepared ointment was stored in the refrigerator (2–8°C) until use.

### 2.3 Rheological characterization of the creams

Rheological measurements were performed by Kinexus Pro Rheometer (Malvern Instruments Ltd, UK) registering the data with rSpace for Kinexus Pro 1.3 software. Semisolid samples were characterized using a cone and plate geometry where the gap for sample placement was 0.15 mm. Rotational measurements of formulations were determined at 25°C controlled with an accuracy of ±0.1°Cby Peltier system of the instrument. In all measurements a cylindrical cover made of stainless steel was placed over the samples, in order to create a closed, saturated volume round the sample and to prevent evaporation of the samples.

The particle size distribution in the five different creams was also measured with a Malvern Mastersizer2000 (Malvern Instruments Ltd, Malvern UK) laser diffraction device. 1 g of sample was dispersed in 10 ml of water, and after 1 minute of Vortex shaking, 1 ml was taken and added to the sample dispenser unit containing 100 mL purified water. The dispenser unit was applied at 1500 RPM.

The multi-channel detector system measured the scattering of red laser light with a wavelength of -632.8 nm, and the evaluation software provided the results after three parallel exposures according to the Fraunhofer method. The average curves were calculated from three parallel measurements.

### 2.4 Skin-on-a-chip diffusion experiments

The skin-on-a-chip microfluidic diffusion chamber is a polydimethylsiloxane-based system in an aluminum frame. Similarly, to the Franz diffusion cells, it contains two compartments, and the membrane or skin sample is placed between them. The major advantage of this technique is the reduction of volumes and required components, membranes, and skins, as discussed in details in our previous papers [38, 39]. The diffusion surface was 0.5 cm^2^, which was separating the cream containing donor chamber and the PPF filled receptor chamber. Contrary to the static Franz diffusion cells, the microfluidic diffusion chamber is a dynamic system, i.e., the PPF flow is continuous in the receptor compartment, ensured by a syringe pump (NE-1000, New Era, Farmingdale, NY, USA), which includes a 5 mL syringe filled with PPF, and running at a flow rate of 4 μL/min during the experiment. The samples were collected every 30 or 15 min for 5,6 or 12 h, depending on the experimental question. After collection, the samples were immediately placed on dry ice and stored at -80°Cuntil the bioanalysis. Skins were provided by mice for 1) psoriatiform dermatitis and 2) for allergic contact dermatitis studies. While rat skins were used for 1) testing the effect of temperature on the degree of transdermal drug diffusion and 2) for investigation of steroidal and non-steroidal anti-inflammatory drugs. In case of thermodynamic study in rats, first, the experiments were performed at room temperature (appr. 23.5°C) regulated with air conditioning, and later repeated at skin temperature (32°C), putting the whole system into an incubator (Steriliser SN160, Memmert GmbH, Schwabach, Germany). Caffeine concentration of the collected samples was determined by NanoDrop spectrophotometer at 273 nm. The anti-inflammatory drugs were determined by LCMS-MS method.

### 2.5 Animal models of psoriasis and allergic contact dermatitis

The C57BL/6J mouse strain was purchased from Charles River Laboratories (Germany) and housed in a conventional animal facility. The animals were provided with unrestricted access to water and fed ad libitum. For the experiments, age-matched male mice were selected. All experiments were approved by the Animal Experimentation Review Board of Semmelweis University and the Government Office for Pest County (Hungary) (Ethical approval number: PE/EA/1967–2/2017) and performed in compliance with the guidelines of the Association for Assessment and Accreditation of Laboratory Animal Care International.

Two days prior to the induction of **psoriasis**, the mice were shaved on their backs with Aesculap trimmer GT420 Isis within an area of 3×2 cm^2^. Also, depilating was implemented two days prior to the experiment and was conducted daily. The mice were treated superficially on the hairless skin every day with 0.0625 g, 5% (m/m) Imiquimod containing Aldara cream (University Pharmacy, Budapest, Hungary). The same amount of vehicle cream was used as a control. For the 24-h treatment group, the mice were treated only on the first day and after that they were euthanized by 5% isoflurane inhalation. Double skin thickness was measured with a microcaliper (Käfer Messuhrenfabrik GmbH & Co. KG, Villingen- Schwenningen, Germany) on the shaved area. Two measured parameters were taken on the area of the scapula, whereas the other measurement was conducted near the sacrum. In addition, erythema and scaling (desquamation) scores were also defined for the observed area (according to [40]). At the end of the experiment the animals were sacrificed by inhalation of 5% v/v isoflurane (in a mixture of 30% oxygen and 70% nitrogen) and spleen was dissected and weighted.

**Allergic contact dermatitis** was induced by topical treatment of the shaved abdominal skin of mice with 100 μL of 3%(m/V) 2,4,6-trinitro-1-chlorobenzene (TNCB, Sigma Aldrich, Budapest, Hungary) dissolved in acetone (Molar Chemicals, Budapest, Hungary) or in case of control animals, with acetone only. After five days, ear thickness was measured with a microcaliper (Kafer), then all mice (even the control ones) were challenged by epicutaneous treatment with 20 μL of 1%(m/V) TNCB on both ears (10 μL on the inner part and 10 on the outer part of the ear). After 24 h, ear thickness was measured again, the mice were terminated by isoflurane over-anesthesia (5% v/v) and ear tissues were used for further investigations [41].

### 2.6 Skin preperation

The skins were prepared as it was described earlier by our group [42–44]. In case of disease models mice were used, while in the investigation of anti-inflammatory drugs and when the effect of temperature was studied, rat skins were applied. In the ex vivo experiments, the abdominal or auricular skins of mice with a body weight of 24.2–31.2g (contact dermatitis) and 21.7–27.0g (psoriasiform dermatitis) or abdominal skins of rats (599–615 g) were applied. The excision of the skins was conducted after over-anesthesia as it is described above. The mouse skins were prepared as described in subsection 2.5, while in case of rats the abdominal skin surface was shaved using an electric shaver and depilated using a depilatory cream (X-Epil, Aveola Kft., Budapest, Hungary). Subsequently, it was washed, wiped dry, and mechanically sensitized by tape stripping with an adhesive tape (BSN Medical GmbH, Hamburg, Germany) with 10 replicates to remove the upper layers of dead keratinocytes. This technique enables to reach detectable concentrations of the test drugs’ in the dermis and hypodermis and finally in the PPF in receptor chamber of the microfluidic device. The double skin thickness of the depilated, tape-stripped skin was measured in the rats similarly to mice. At the excision, after the first cutting with blunt Metzen scissors, the connective tissue, the adipose tissue, and the blood vessels were carefully removed from the rat abdominal skins.

For the skin-on-a-chip studies, full-thickness skin samples were made from the previously prepared skin explants with a diameter of 18 mm using skin-punches (Stubai GmbH, Fulpmes, Austria). The excised samples were then wrapped in aluminum foil and stored in a deep freezer at -80°C until the diffusion experiments were conducted. On the day of the diffusion study, before initiation of the experiment the skin samples were allowed to reach room temperature for 30 minutes.

### 2.7 Mathematical modeling and parameter estimation

The compartmental mathematical model we used is a slightly modified 3-compartment form of the one described in [1] (Eqs. (55)–(57)). We combine diffusive and convective transport (Eqs. (3) and (8)) and write the discretized system as

$$\begin{array}{cc} \frac{\text{d}x_{1}}{\text{d}t} & =k_{in}+k_{d}x_{2}-\left(k_{d}+k_{c}\right)x_{1},\\ \frac{\text{d}x_{2}}{\text{d}t} & =\left(k_{d}+k_{c}\right)x_{1}+k_{d}x_{3}-\left(2k_{d}+k_{c}\right)x_{2},\\ \frac{\text{d}x_{3}}{\text{d}t} & =\left(k_{d}+k_{c}\right)x_{2}-\left(k_{d}+k_{out}\right)x_{3}. \end{array}$$ (1)

**Fig 1:**
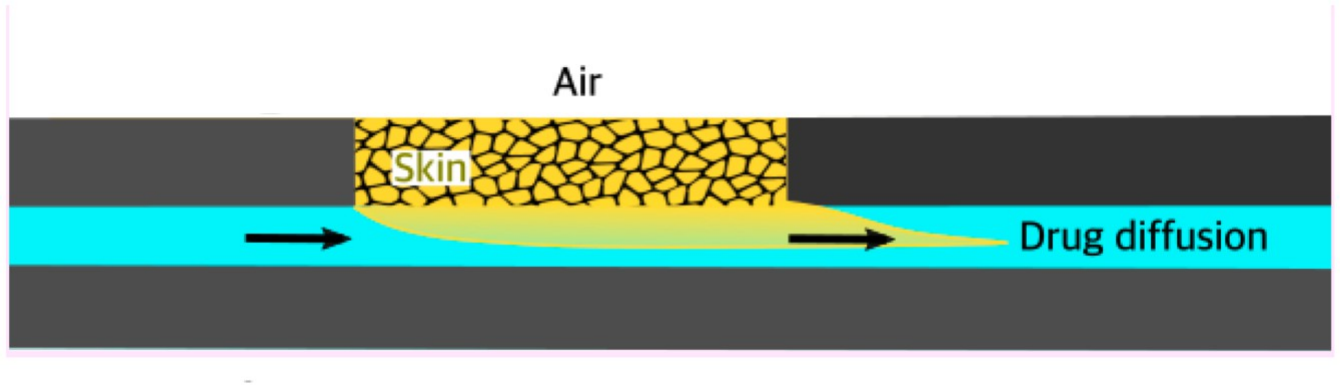
Penetration of topically applied drug formulation through the skin into the perfusion fluid of skin-on-a-chip microfluidic diffusion chamber (This figure was prepared by Prof. Shanmugam Dhinakaran Indore, India).

Here the state variables *x*_*i*_ represent the concentration of the compartments. The unknown positive parameters to be estimated are the inflow, the diffusion coefficient, the convection coefficient and the outflow, respectively denoted as *k*_*in*_, *k*_*d*_, *k*_*c*_ and *k*_*out*_ (see, also Fig 1). The only measured concentration is *y* = *x*_3_.

It is visible that the model (1) is linear both in terms of state variables and parameters. Therefore, its structural identifiability can be easily checked. The DAISY toolbox using differential algebra [45] was used for this purpose, and it was concluded that the model is globally structurally identifiable with respect to the four unknown parameters even if the initial conditions of the non-measured variables are unknown. The non-measured concentrations can be eliminated from the model as follows:

$$\begin{array}{c} y^{\left(3\right)}\left(t\right)+\theta_{1}y^{\left(2\right)}\left(t\right)+\theta_{2}y^{\prime }\left(t\right)+\theta_{3}y\left(t\right)+\theta_{4}=0 \end{array}$$ (2)

where

$$\begin{array}{c} \theta_{1}=2k_{c}+4k_{d}+k_{out} \end{array}$$ (3)

$$\begin{array}{c} \theta_{2}=k_{c}^{2}+3k_{c}k_{d}+2k_{out}k_{c}+3k_{d}^{2}+3k_{out}k_{d} \end{array}$$ (4)

$$\begin{array}{c} \theta_{3}=k_{out}k_{c}^{2}+2k_{out}k_{c}k_{d}+k_{out}k_{d}^{2} \end{array}$$ (5)

$$\begin{array}{c} \theta_{4}=-k_{in}{\left(k_{c}+k_{d}\right)}^{2} \end{array}$$ (6)

It is easy to see from Eq (2) that the eliminated model is a regression model in the transformed parameters *θ*_1_, …, *θ*_4_. Therefore, it is at least theoretically possible to uniquely compute the four unknown parameters from the measurements of *y* = *x*_3_.

The parameters of the model (1) were estimated using the algorithm [46] implemented in the fmincon function of Matlab. The following objective function was minimized with respect to the parameters *k*_*c*_, *k*_*d*_, *k*_*in*_, and *k*_*out*_:

$$\begin{array}{c} V=\frac{\|y_{m}-y_{c}{\|}_{2}}{\|y_{m}{\|}_{2}} \end{array}$$ (7)

where *y*_*m*_ is the measured and *y*_*c*_ is the computed concentration, respectively, and ‖ ⋅ ‖_2_ denotes the 2-norm. To avoid local minima, several optimization runs were performed for each data series started from 200 different initial conditions. In agreement with the identifiability results, all the optimizations showed good convergence and unique minima.

## 3 Results and discussion

This section contains the measurement and calibration results for the studied scenarios. The detailed results of parameter estimation can be found in S1 Table.

### 3.1 Viscosity and particle size distribution of the creams

**Fig 2:**
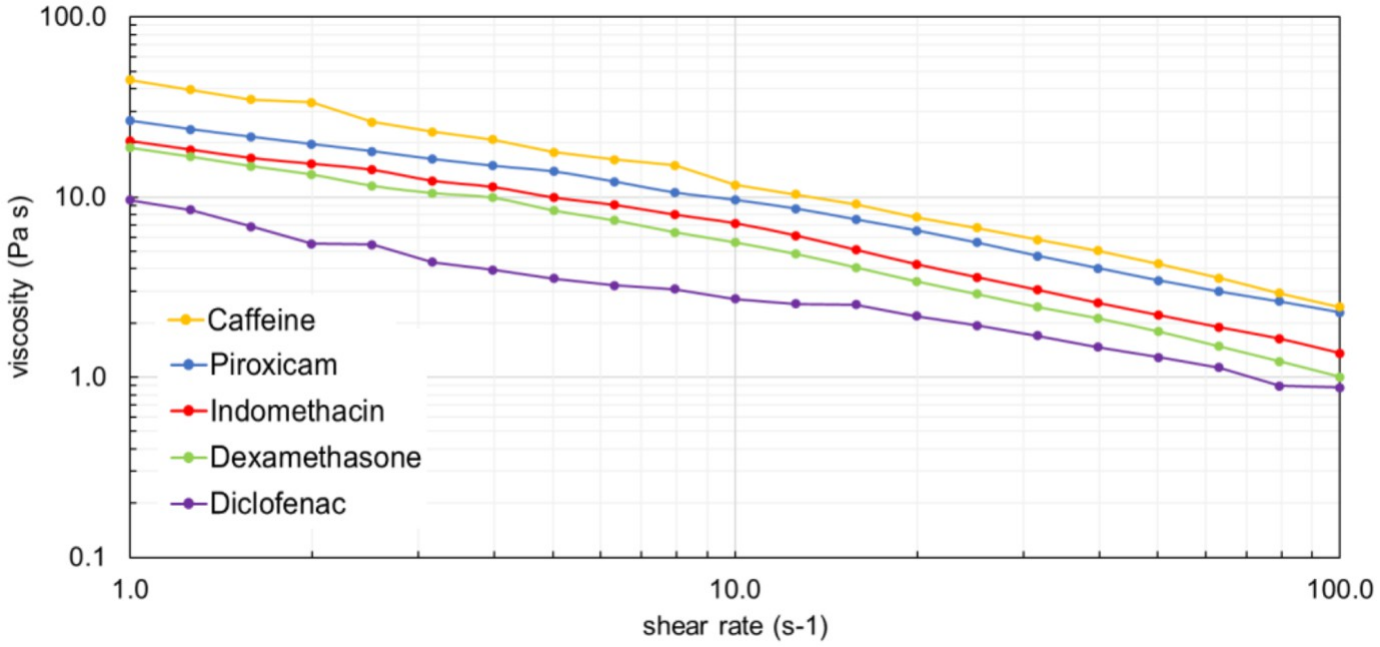
Viscosity curves of creams containing 2% of active ingredient. All active compounds were used in 2% in O/W suspension-cream formulation.

**Fig 3:**
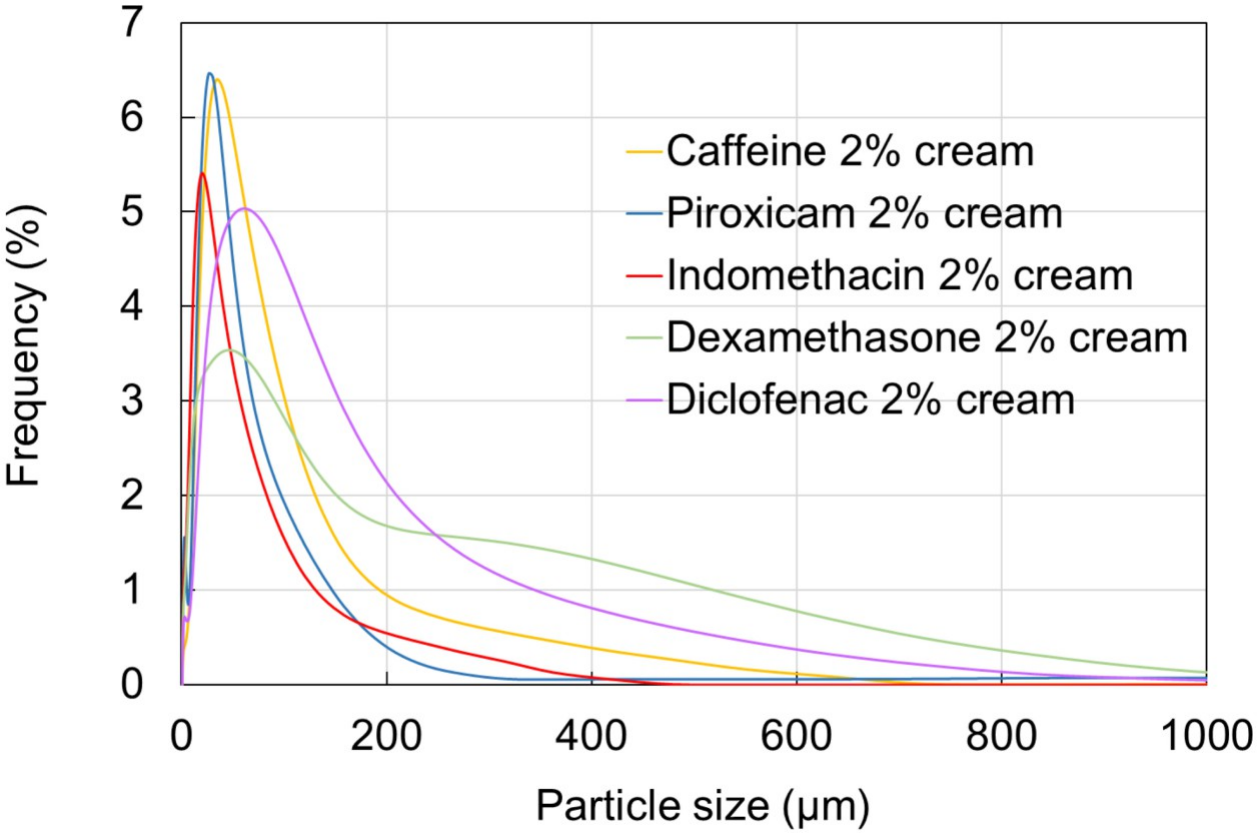
Particle size distribution of creams containing 2% active ingredient.

Before the diffusion experiments the five drug-containing creams were subjected to rheological investigations and testing particle size distribution. The viscosity curves are shown in Fig 2 and the particle size frequency is presented in Fig 3.

Caffeine cream presented the highest viscosity and the smallest but quite homogeneous particle size distribution. On the other hand, diclofenac cream was the less viscous and showed a wider distribution of particle size. Dexamethasone cream was also inhomogeneous in regard of particle size.

### 3.2 Transdermal delivery of caffeine at ambient (23.5°C) and skin temperature (32°C)

**Fig 4:**
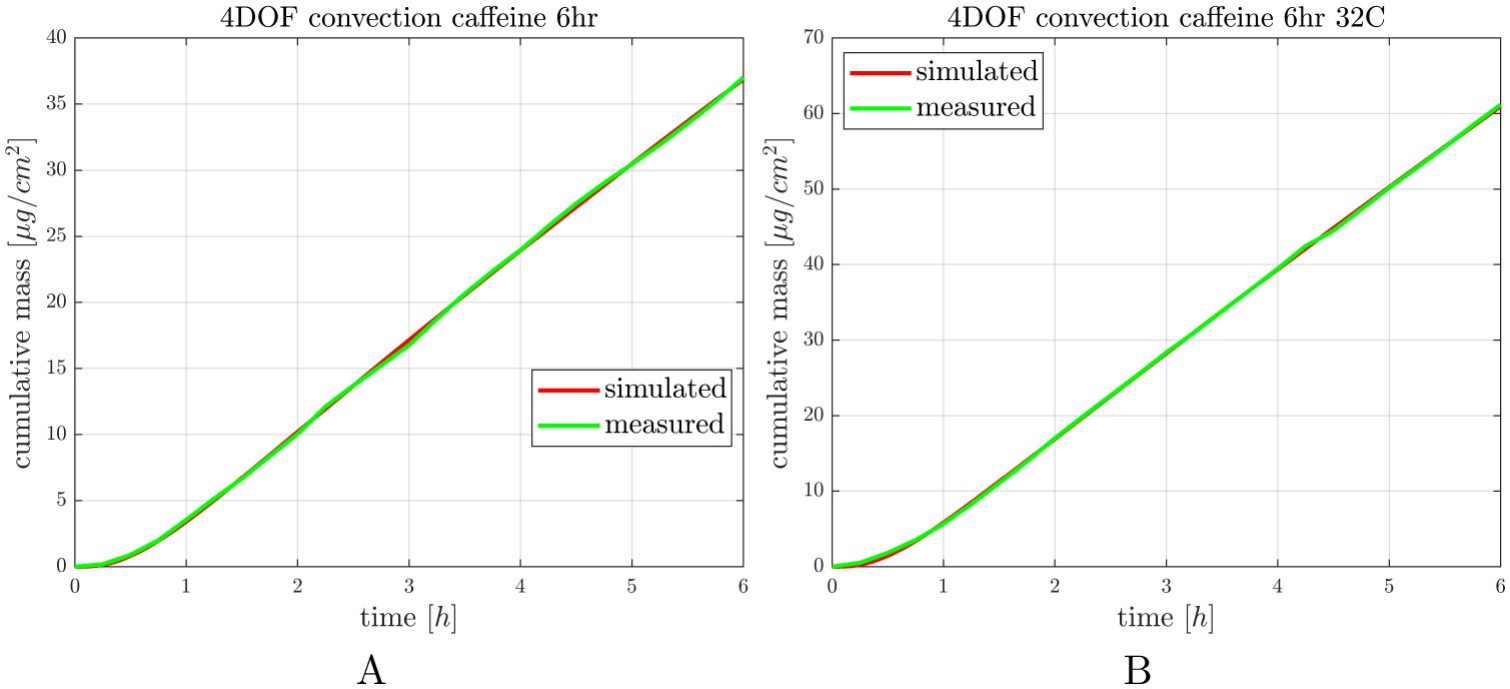
Caffeine penetration through the ex vivo mouse skins in “skin-on-a-chip” microfluidic device at room temperature (panel A) and skin temperature (panel B). The curves show the average of 3 experiments.

First transdermal delivery of caffeine was tested in healthy mouse skin in dynamic skin-on-a-chip device at room temperature and skin temperature. The study was conducted in three replicates. The concentration-time profiles were plotted and the total penetrated amount of caffeine was normalized for the diffusion surface. After the in vitro experiments in silico simulations were also performed. The measured and calculated data are shown in Fig 4A and 4B, see also S1 Fig.

### 3.3 Transdermal delivery of caffeine with prolonged data collection on ambient temperature

**Fig 5:**
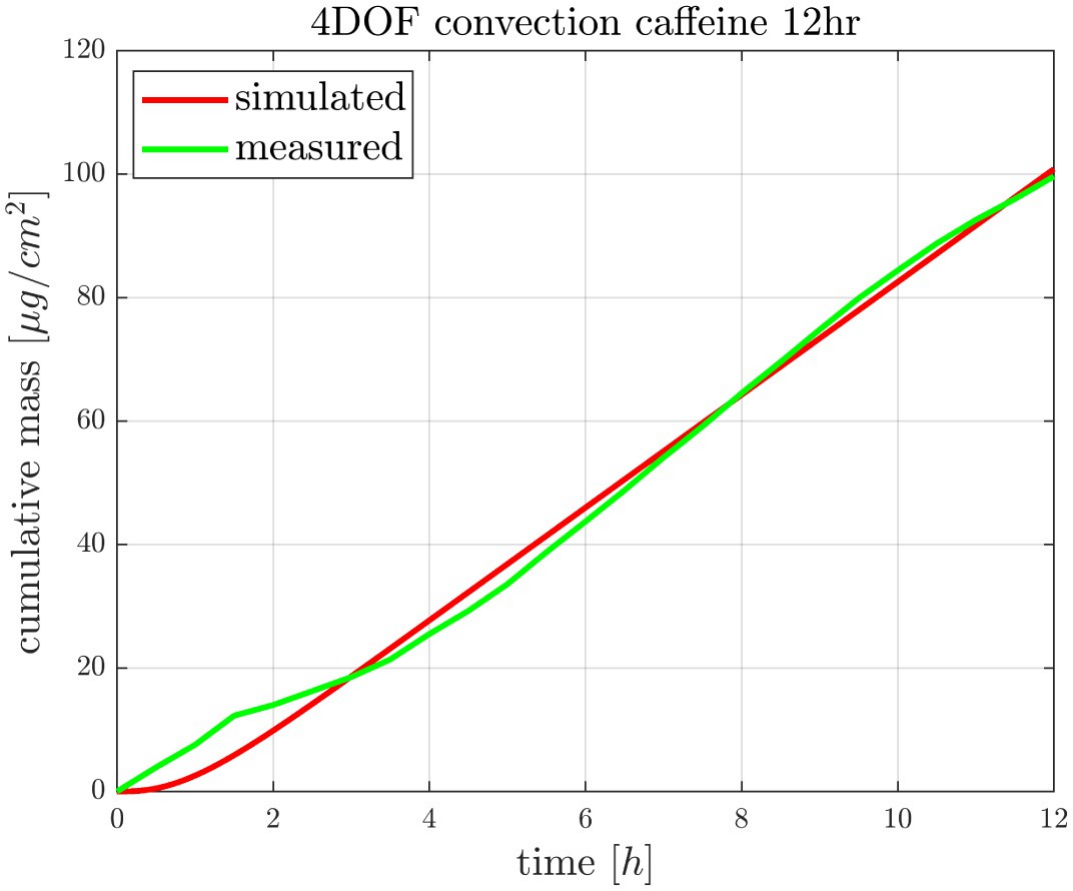
Caffeine penetration through the ex vivo mouse skins in “skin-on-a-chip” microfluidic device at room temperature, monitored with extended exposure time (12h). The curves show the average of 3 experiments.

In the second series of experiments the sample collection time was extended in that hope, that after 12 h exposition time a steady state diffusion can be achieved and the curves reach a saturated characteristics. This study was performed only at room temperature. As it can be seen in Fig 5, the penetrated drug amount still showed a monotonous increase with time at the end of observation period. These results raise the question, whether the 4 *μ*l/min flow rate is the optimum or should be modified to make a physiologically more relevant condition. The other question is that whether the applied cream volume is appropriate or perhaps too big, to get more realistic outcome. These questions will be analyzed in our next project by computational fluid dynamics method. However, the simulation data and the measured data of the current study show a very good fit in the applied conditions and provide a nearly linear profile.

### 3.4 Transdermal delivery of steroidal (SAID) and non-steroidal anti-inflammatory drugs (NSAIDs)

In the next experimental setup dexamethasone, piroxicam, indomethacin and diclofenac creams were tested for penetration of the active ingredients across the dermal barrier. The physicochemical properties of the anti-inflammatory drugs are shown below in Table 1.

**Table 1:** Physicochemical properties of anti-inflammatory drugs used in the experiments and for simulation.

Compound	MW	logP	Chemical formula
Dexamethasone acetate	434.50	2.6	C_24_H_31_FO_6_
Piroxicam	331.35	2.2	C_15_H_13_N_3_O_4_S
Indomethacin	357.79	4.25	C_19_H_16_ClNO_4_
Diclofenac	318.13	4.75	C_14_H_11_Cl_2_NO_2_

**Fig 6:**
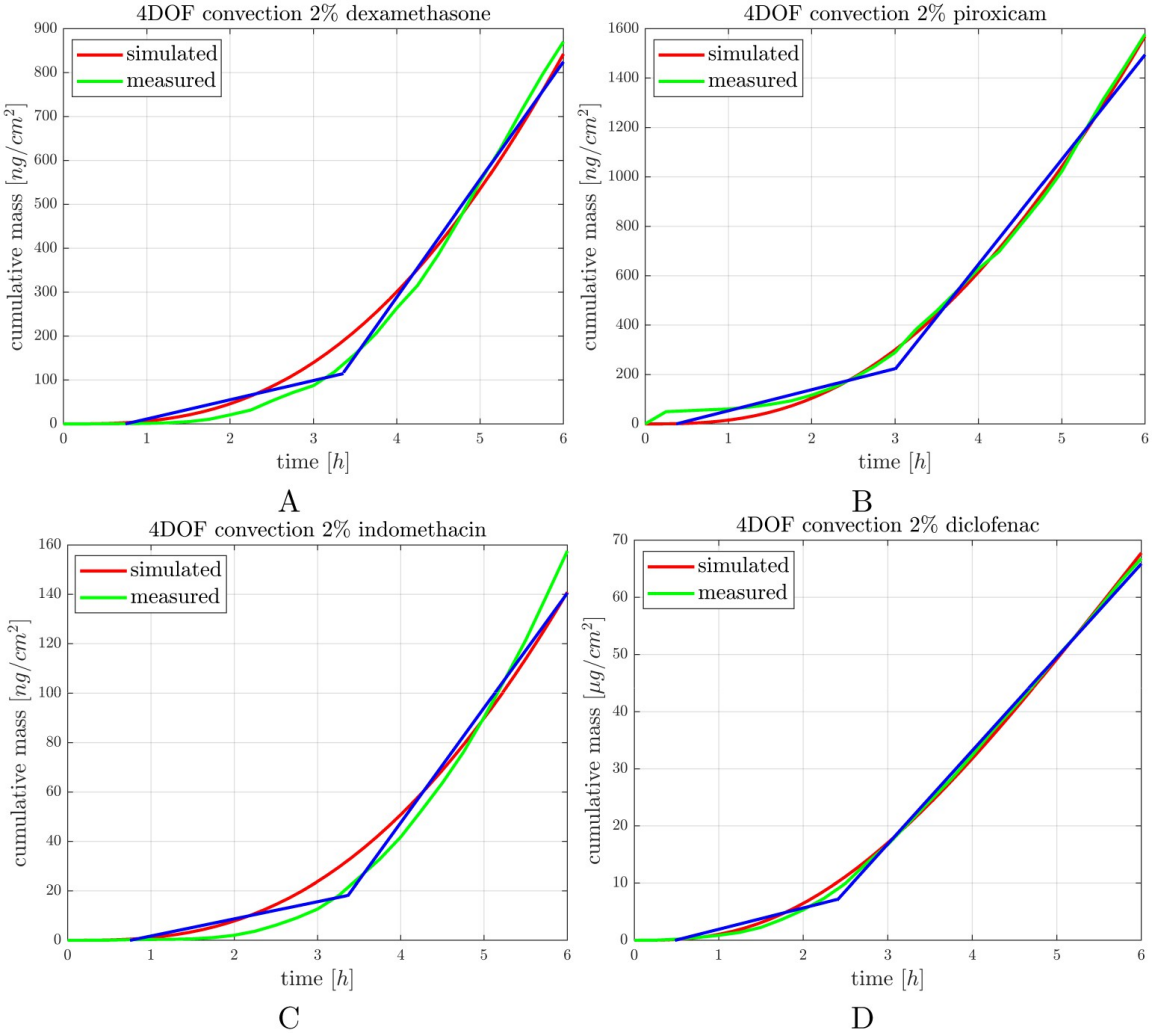
Penetration of anti-inflammatory drug formulations through the ex vivo mouse skins in “skin-on-a-chip” microfluidic device at room temperature, monitored during 6 h exposure time. Blue line: regression lines showing the slow and rapid phases of the process. The curves show the average of 3 experiments.

It can be seen in Fig 2, that diclofenac cream showed the lowest viscosity and based on the penetration study (Fig 6) it penetrated in the lowest amount through the diffusion surface presenting a consistent transport dynamics. Out of the four anti-inflammatory drugs tested, piroxicam cream had the highest viscosity, and most homogenous particle size distribution (Figs 2 and 3). Also, piroxicam penetrated in the highest amount through the ex vivo skin within the 6 h observation period (Fig 6). Furthermore, the penetration kinetics of piroxicam was slower at the first 3 h, and accelerated after it, in the rest of the time. Dexamethasone and indomethacin penetrated in a smaller degree and showed also a slower initial phase, followed by faster absorption profile between 4–6 h, indicating a release period from the formulation and a diffusion period after the penetration into the deeper layers of the skin (Fig 6, S2 Fig).

### 3.5 Skin penetration of caffeine in psoriasiform dermatitis

**Fig 7:**
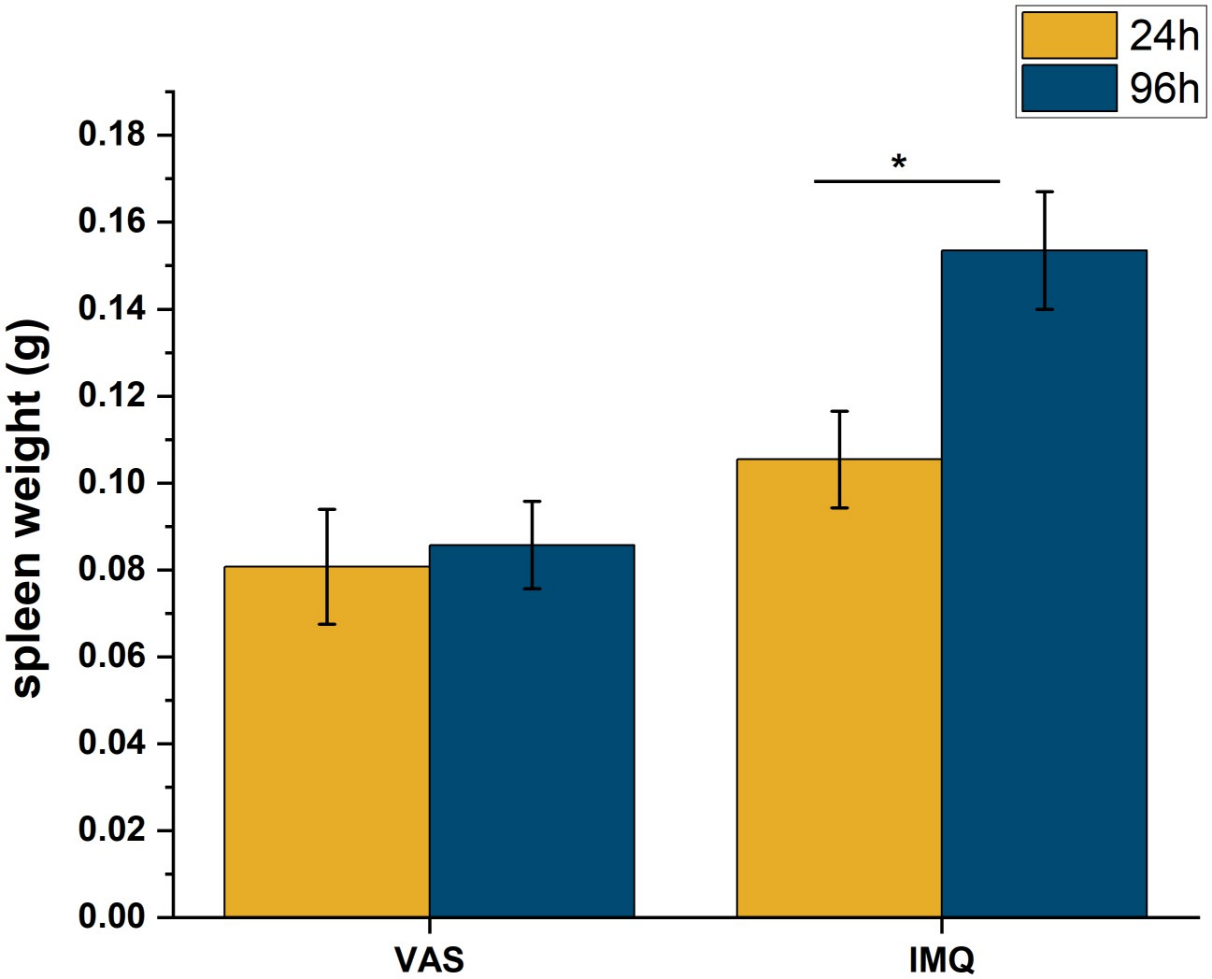
Spleen weights in control (vaseline) and psoriatic (IMQ) mice 24 and 96 h after the induction of the psoriasiform dermatitis. Means ±SEM. *: *p* < 0.05, *n* = 3/group.

In the previous studies healthy mouse skins were integrated into the skin-on-a-chip devices. On the next experiments the ex vivo skin preparations were acquired from either psoriatic or allergic mice. To provide evidence, that the induction of psoriasis with imiquimod (IMQ) cream was successful, the spleen weight was measured at the end of the experiments after termination of the animals. Wild type mice were used and divided into control (vaseline treated) and psoriatic (imiquimod treated) groups at 24 h and 96 h evaluation time. The results are presented in Fig 7.

**Fig 8:**
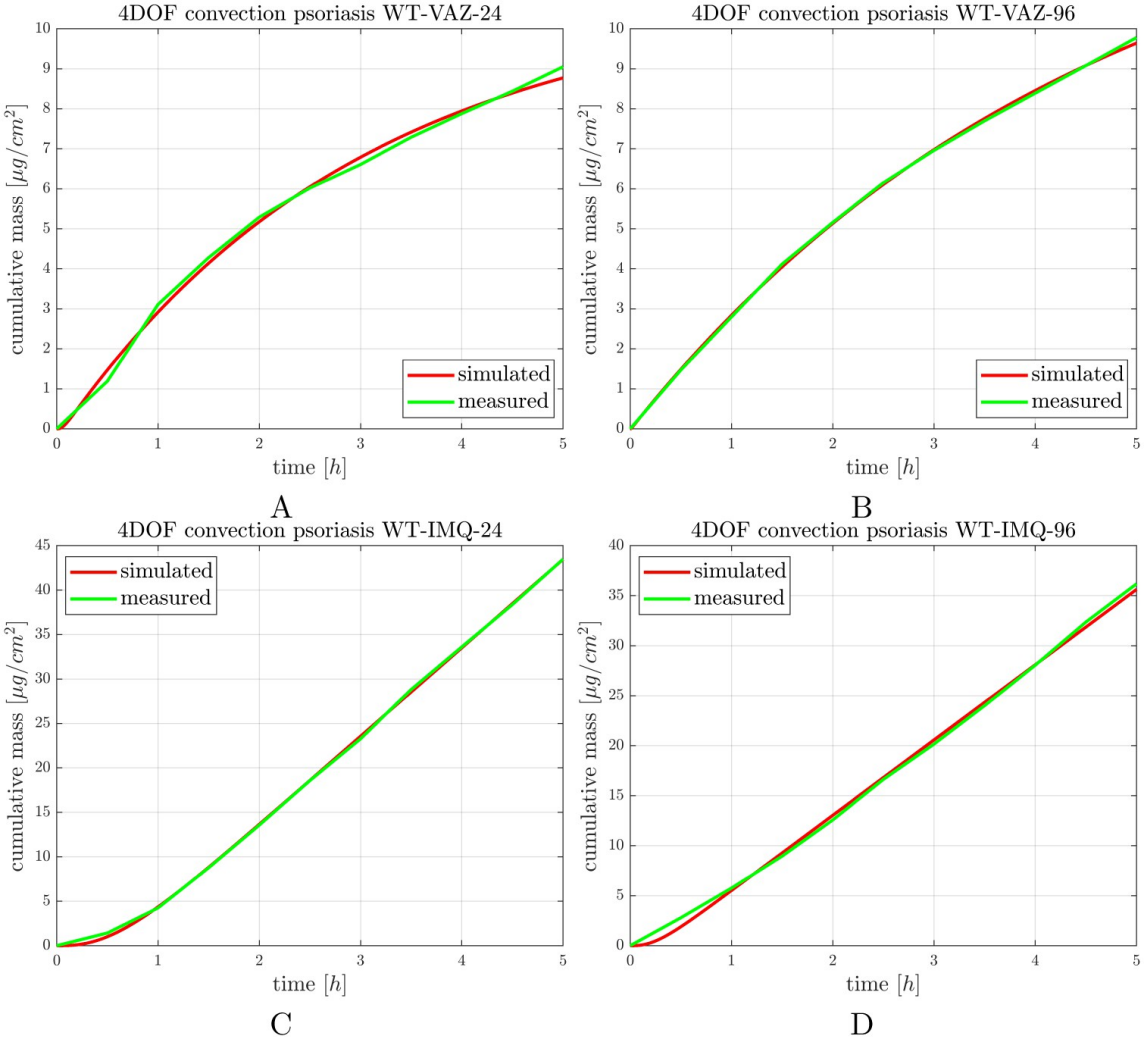
Caffeine penetration through excised mouse dorsal skins in A,B: control (vaseline) and C, D: psoriatic (IMQ) mice 24 and 96 h after the induction of psoriatiform dermatitis. The curves are average curves of 3 experiments.

IMQ induced desintegration of the dermal and epidermal barrier by increasing the skin permeability for caffeine at already 24 h and also at 96 h after the exposition. Compared to the time-matched controls the permeability increased more than 4 times at 24 h, and more than 3.5 times at 96 h. The inflammation caused damage on the full thickness of the skin which can be established based on linear characteristic of the cummulative mass-time profiles (Fig 8, S3 Fig) We can not separate a first release phase and a second absorption phase in the diseased skins with psoriasiform dermatitis.

### 3.6 Skin penetration of caffeine in allergic contact dermatitis

**Fig 9:**
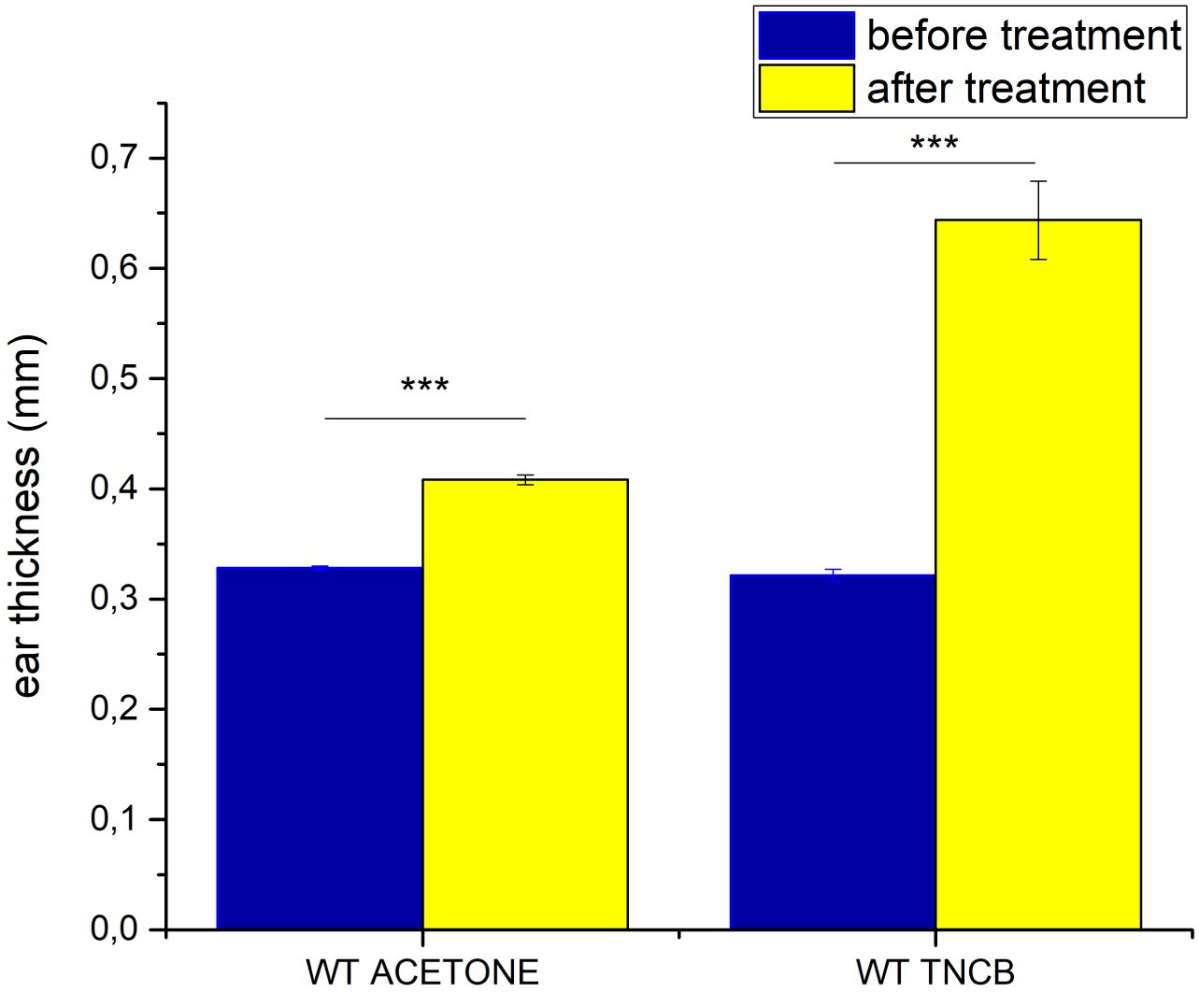
Ear thickness in control (acetone) and allergic (TNCB) mice before and after the second exposition to the inducers. Means ±SEM. ***: *p* < 0.0001, *n* = 3/group.

**Fig 10:**
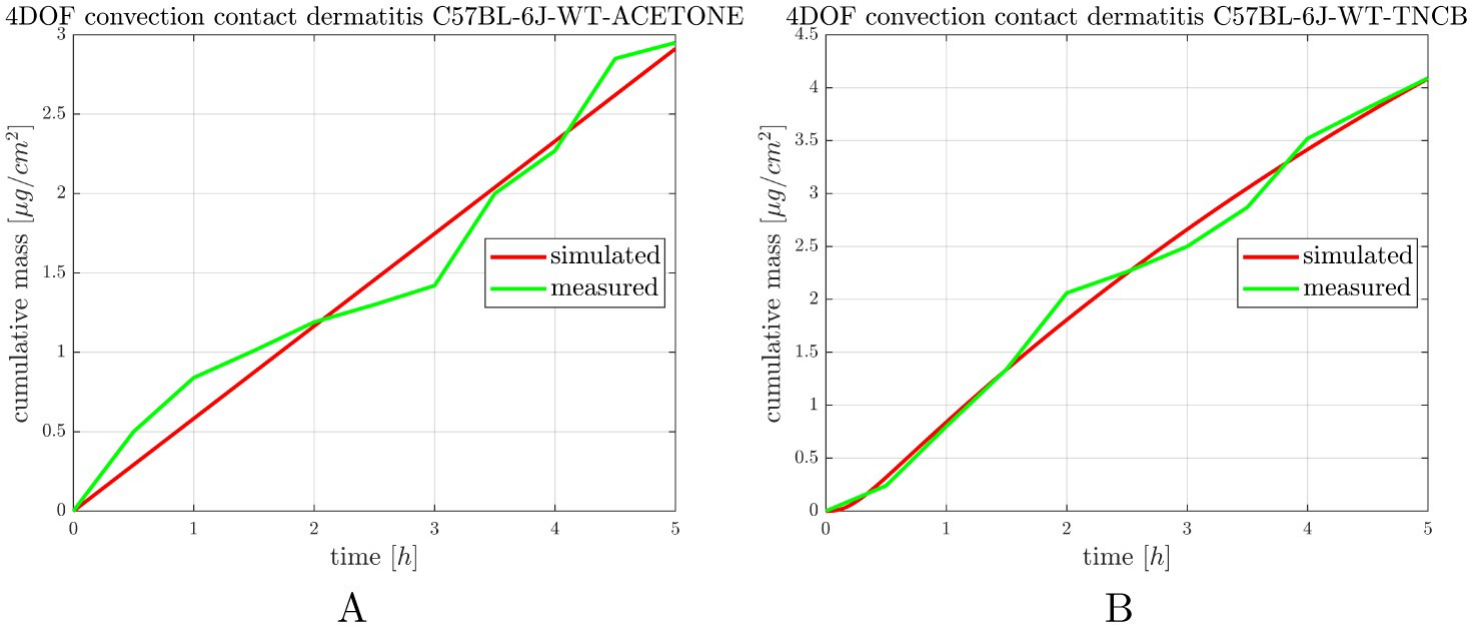
Caffeine penetration through the mouse ear skin barrier (auricular tissue) in A: control (acetone treated) and B: allergic (TNCB treated) mice. The curves are average curves of 3 experiments.

To prove that the induction of allergic contact dermatitis was successful, the thickness of the ears was measured before and after the second exposure (elicitation) to the hapten (TNCB) or the vehicle (acetone) (Fig 9). In this case the auricular tissues were integrated to the skin-on-a-chip devices and the permeability for caffeine was tested on the ears (Fig 10, S4 Fig).

The topical treatment with acetone alone induced a moderate swelling of the ears, while TNCB treatment resulted in statistically significant edema formation (increased thickness) in the ears (Fig 9).

The allergic contact dermatitis induced a 30% increase in the permeability of the ears of mice. The penetration characteristic showed single-phase, consistently increasing, linear profile in all groups of animals (Fig 10). The simulation fits well to the curves, but demonstrates less variability and a smoother kinetics, than the measured values. The variability of the measured data can be explained by the swelling and edemotous properties of the ears, which did not appear evenly over the entire tissue.

The exclusion criteria for animals and measured data are given in Table 2.

**Table 2:** Summary of exclusion criteria applied before evaluation and simulation of the measured data. Animals and measurement values below the minimum and above the maximum were excluded. The criteria were applied a priori in the case of animal selection, and a posteriori after having the experimental results in the case of concentration measurements.

criteria	minimum value	maximum value
Mouse body weight	20 g	32 g
Rat body weight	550 g	620 g
Caffeine cumulative mass 6h RT	20 μg/cm^2^	50 μg/cm^2^
Caffeine cumulative mass 6h 32oC	40 μg/cm^2^	75 μg/cm^2^
Caffeine cumulative mass 12h RT	80 μg/cm^2^	120 μg/cm^2^
Dexamethasone cumulative mass 6h	600 ng/cm^2^	1000 ng/cm^2^
Indomethacin cumulative mass 6h	100 ng/cm^2^	200 ng/cm^2^
Piroxicam cumulative mass 6h	1200 ng/cm^2^	2000 ng/cm^2^
Diclofenac cumulative mass 6h	50 μg/cm^2^	100 μg/cm^2^

## 4 Conclusions

In recent years more and more attention has been paid to the in vitro and in silico technologies to replace and reduce the number of experimental animals sacrificed in pharmaceutical and bio-medicinal research. In accordance with the general requirements several organ-on-a-chip models were developed to mimic the physiological barriers of the body (for a review see, [47]). However, the majority of these devices are still in a proof of concept phase and not utilized routinely. The aim of our current research was to create a relevant, low variability, reproducible and cost effective in vitro/ex vivo system based on a skin-on-a-chip microfluidic device and to produce a reliable mathematical model to simulate and predict topical drug penetration across the dermal barrier. It is known that physiologically based dermal absorption models can efficiently support the bioavailability assessment of topical drugs [48]. Therefore, we proposed a simple compartmental ODE model to give insight into the temporal profiles of permeant absorption. The structural identifiability of the model was checked, showing that the model parameters can be uniquely determined from sufficiently informative measurements. Our in silico model was calibrated using caffeine as a hydrophilic model drug under different conditions such as various temperatures, short and long-term exposition time, furthermore healthy, psoriatic and allergic situations. To test whether the in silico model can be adapted to different chemical entities, various anti-inflammatory molecules formulated in the same basic ointments were also assessed. For physical evaluation of the creams with different active ingredients rheological investigation and particle size distribution studies were conducted. Our results indicate the mathematical model developed is able to provide an appropriate tool to simulate the diffusion dynamics of percutaneous drugs in our single channel microfluidic device. Additionally, the calculation models considered in this article may be applicable not only for the tested compounds but also to other drugs. Computer analysis of experimentally determined diffusion data in the manner described may provide information concerning the relative importance of various routes of penetration for molecules with different physicochemical characteristics and also for the barrier function of the skin in inflammatory conditions. In our case a three-compartmental model was applied but if the question is more complex, then the number of compartments integrated into the model can be increased. In certain cases the test substances should only penetrate to the superficial layer of the skin (cosmetics), or to the appendages (anti-acne drugs, antiperspirants) or to the viable epidermis (anti-histamines, anesthetics) or to more deeper layers of dermis (nicotine, scopolamine or hormone containing formulations) or even through the entire skin to the joint or the muscle (anti-inflammatory drugs, analgesics) [21]. Considering all these aspects, the utilization or further modification of our model greatly depends on the therapeutic goal and the properties of the applied topical products. Further work will be focused on developing and applying more detailed models for improved spatio-temporal description of concentration profiles as well as on optimal experiment design.

## Supporting information

S1 Table Parameter estimation results. The estimated parameter values (*k*_*in*_, *k*_*d*_, *k*_*c*_, *k*_*out*_) and the correspoonding minimum objective function values (*V*_*min*_) of the model.
(PDF)

S1 Fig Caffeine penetration through the rat skin in skin-on-a-chip diffusion chamber. Panel A and C at ambient temperature (23.5°C), Panel B at skin temperature (32°C). *n* = 3, means ±SEM.
(PDF)

S2 Fig Transdermal penetration of anti-inflammatory drugs through the rat skin in skin-on-a-chip diffusion chamber at 23.5°C. *n* = 3, means ±SEM.
(PDF)

S3 Fig Caffeine penetration through the mouse skin in skin-on-a-chip diffusion chamber. Panels A and B: control animals (vaseline treated), Panels C and D: psoriatic animals (imiquimod treated). *n* = 3, means ±SEM.
(PDF)

S4 Fig Caffeine penetration through the mouse skin in skin-on-a-chip diffusion chamber. Panel A: control animals (acetone treated), Panel B: allergic contact dermatitis (TNCB treated). *n* = 3, means ±SEM.
(PDF)
