# Supplementary material for: Mathematical modeling of transdermal delivery of topical drug formulations in a dynamic microfluidic diffusion chamber in health and disease
Source: PLoS One. 2024 Apr 11;19(4):e0299501. doi: 10.1371/journal.pone.0299501 (PMC11008853; doi:10.1371/journal.pone.0299501)
Supplement: S1 Table — The estimated parameter values (kin, kd, kc, kout) and the correspoonding minimum objective function values (Vmin) of the model. (PDF) [file pone.0299501.s001.pdf]

S1 Table.

Case	Figure	k_{in}	k_d	k_c	k_{out}	V_{min}
caffeine 6hr	4.A	7.4371	0.0336	3.3778	0.0293	0.0066
caffeine 6hr 32 ℃	4.B	11.8667	0.0319	3.6637	0.0193	0.0048
caffeine 12hr	5	9.2316	0.0198	2.0409	4.2272E-05	0.0449
2% dexamethasone	6.A	874.9814	3.4005E-05	0.2219	3.9239E-05	0.0935
2% piroxicam	6.B	821.8726	1.2034E-05	0.37762	1.58662E-05	0.0392
2% indomethacin	6.C	111.8016	6.457E-04	0.2690	6.4284E-04	0.1383
2% diclofenac	6.D	52.0705	0.0279	0.3681	0.2231	0.0202
psoriasis WT-VAZ-24	8.A	3.6499	0.1206	30.6461	0.3365	0.0216
psoriasis WT-VAZ-96	8.B	3.1878	0.2150	261.0848	0.2207	0.0071
psoriasis WT-IMQ-24	8.C	10.0399	0.0272	3.1361	3.0377E-04	0.0081
psoriasis WT-IMQ-96	8.D	7.5925	0.0637	7.4099	3.4593E-05	0.0222
contact dermatitis C57BL-6J-WT-ACETONE	10.A	0.5846	1.8494	1017.2059	7.3805E-04	0.0954
contact dermatitis C57BL-6J-WT-TNCB	10.C	1.1352	0.0347	9.0611	0.1236	0.0406
