# Supplementary figures and images for: Mathematical modeling of transdermal delivery of topical drug formulations in a dynamic microfluidic diffusion chamber in health and disease
Source: PLoS One. 2024 Apr 11;19(4):e0299501. doi: 10.1371/journal.pone.0299501 (PMC11008853; doi:10.1371/journal.pone.0299501)

A

caffeine 6h

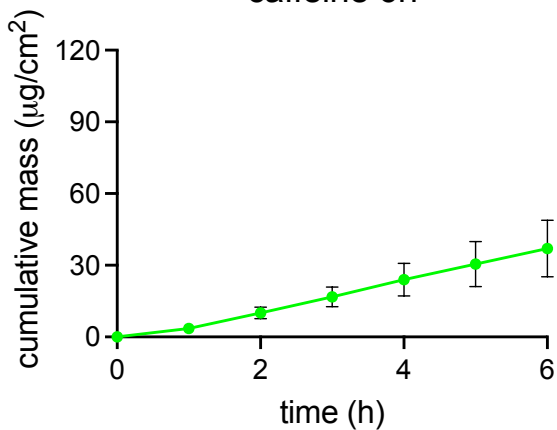

B

caffeine 6h 32°C

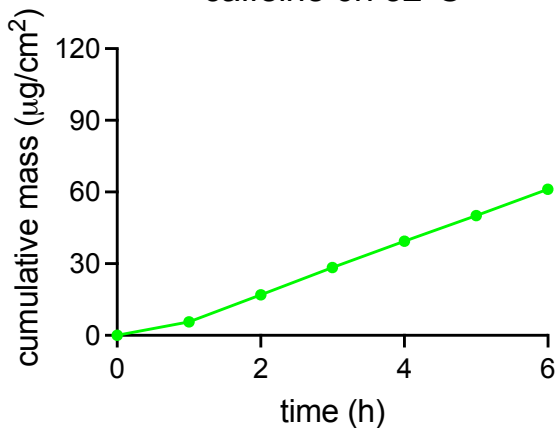

C

caffeine 12h

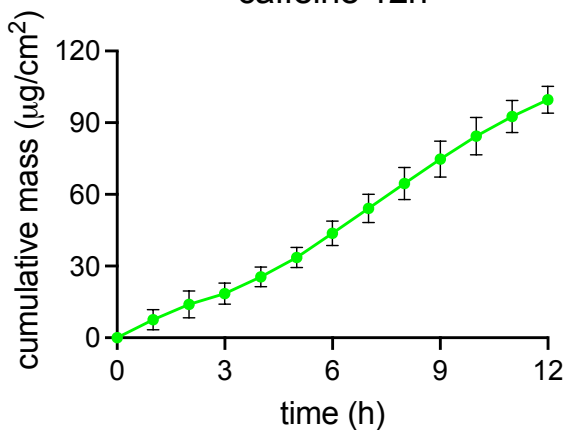

Supplement: S1 Fig — Panel A and C at ambient temperature (23.5°C), Panel B at skin temperature (32°C). n = 3, means ±SEM. (PDF) [file pone.0299501.s002.pdf]

A

dexamethasone

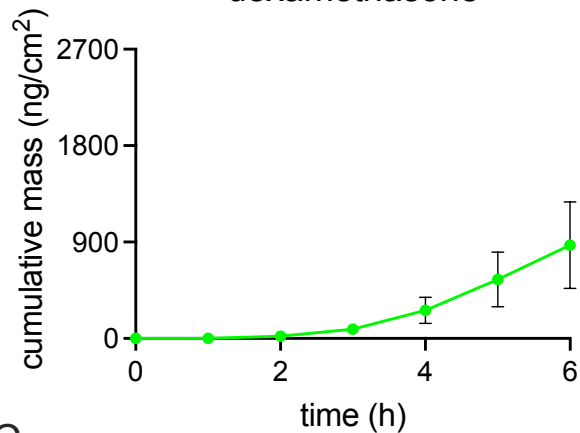

B

diclofenac

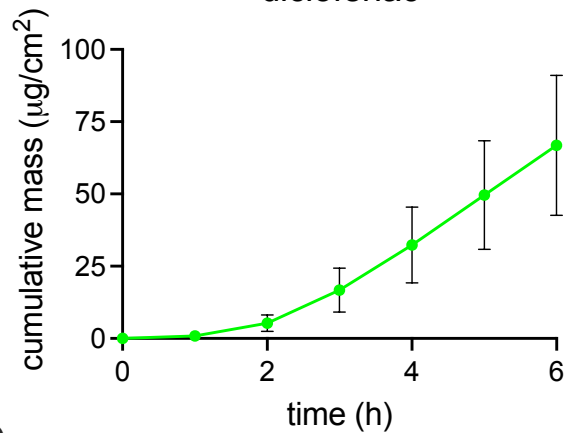

C

indomethacin

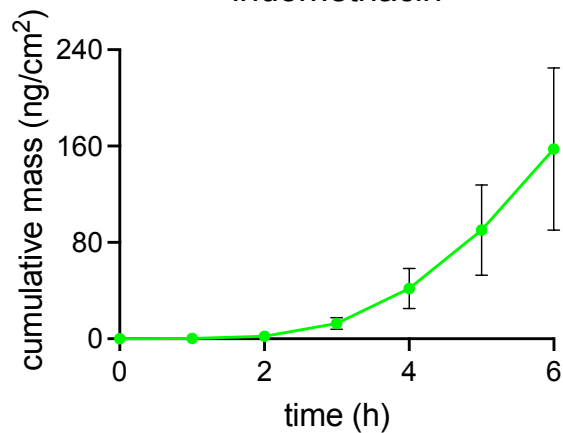

D

piroxicam

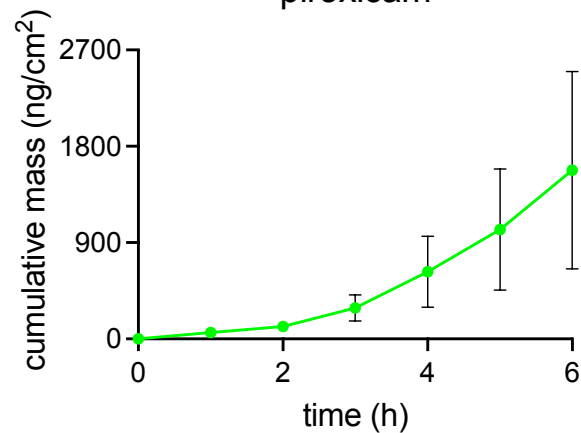

Supplement: S2 Fig — n = 3, means ±SEM. (PDF) [file pone.0299501.s003.pdf]

A

psoriasis - VAS - 24h

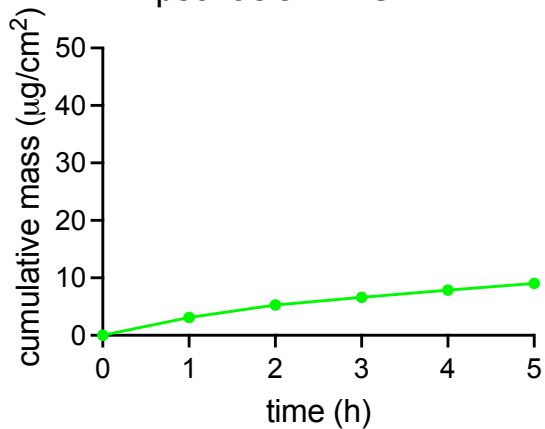

B

psoriasis - VAS - 96h

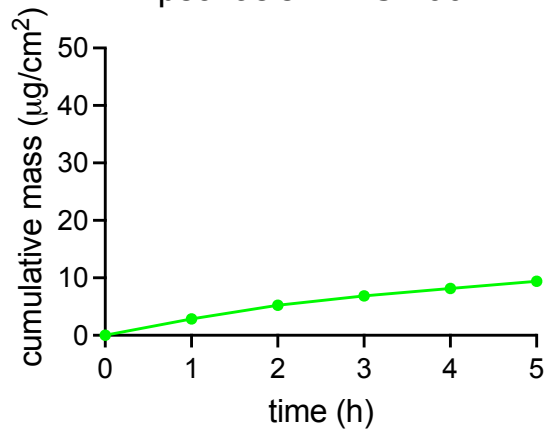

C

psoriasis - IMQ - 24h

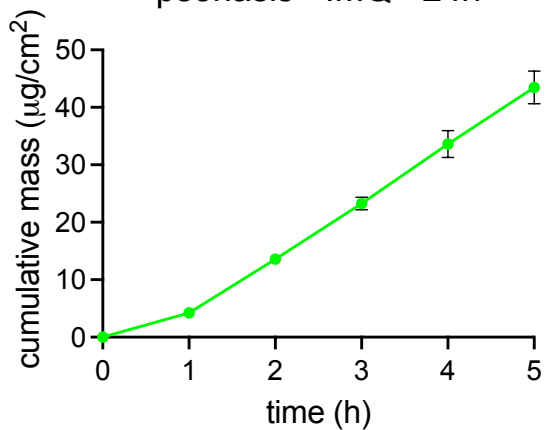

D

psoriasis - IMQ - 96h

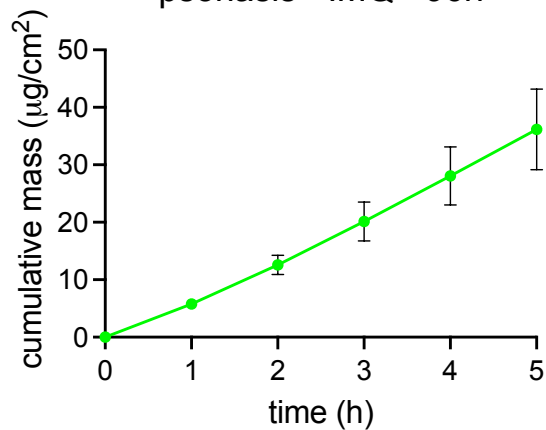

Supplement: S3 Fig — Panels A and B: control animals (vaseline treated), Panels C and D: psoriatic animals (imiquimod treated). n = 3, means ±SEM. (PDF) [file pone.0299501.s004.pdf]

A

contact dermatitis - acetone

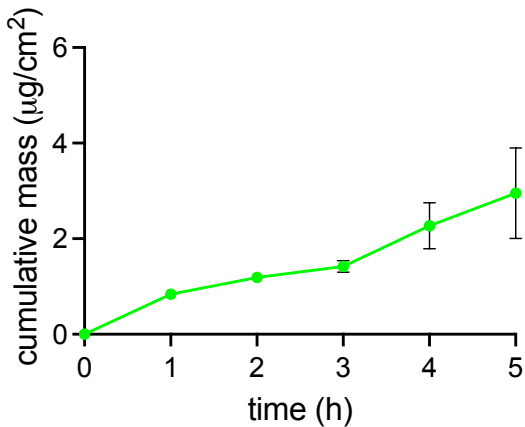

B

contact dermatitis - TNCB

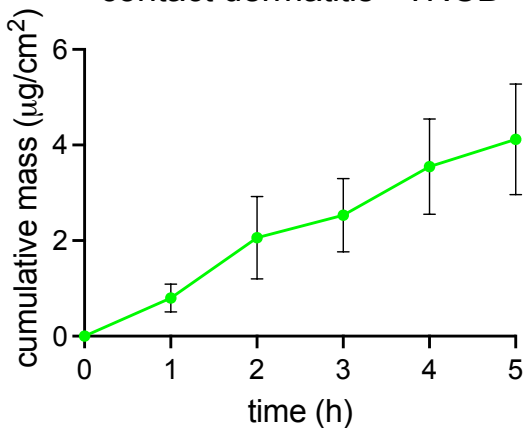

Supplement: S4 Fig — Panel A: control animals (acetone treated), Panel B: allergic contact dermatitis (TNCB treated). n = 3, means ±SEM. (PDF) [file pone.0299501.s005.pdf]
